# Short-Term Memory Affects Color Perception in Context

**DOI:** 10.1371/journal.pone.0086488

**Published:** 2014-01-27

**Authors:** Maria Olkkonen, Sarah R. Allred

**Affiliations:** Department of Psychology, Rutgers – The State University of New Jersey, Camden, New Jersey, United States of America; Radboud University Nijmegen, The Netherlands

## Abstract

Color-based object selection — for instance, looking for ripe tomatoes in the market — places demands on both perceptual and memory processes: it is necessary to form a stable perceptual estimate of surface color from a variable visual signal, as well as to retain multiple perceptual estimates in memory while comparing objects. Nevertheless, perceptual and memory processes in the color domain are generally studied in separate research programs with the assumption that they are independent. Here, we demonstrate a strong failure of independence between color perception and memory: the effect of context on color appearance is substantially weakened by a short retention interval between a reference and test stimulus. This somewhat counterintuitive result is consistent with Bayesian estimation: as the precision of the representation of the reference surface and its context decays in memory, prior information gains more weight, causing the retained percepts to be drawn toward prior information about surface and context color. This interaction implies that to fully understand information processing in real-world color tasks, perception and memory need to be considered jointly.

## Introduction

**Figure 1:**
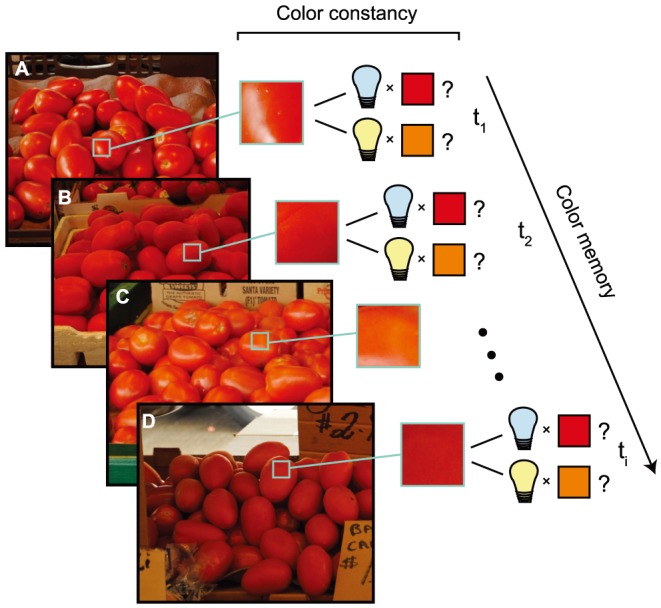
Real-world color tasks — for instance, looking for ripe fruit — pose both perceptual and memory demands. In this example, the key perceptual challenge is to perceive the surface color (perceptual correlate of reflectance) of the tomatoes accurately in each set despite illumination differences within and between sets. This is challenging for vision because many different combinations of surface reflectance and illumination can cause the same light impinging on the eye (illustrated here for sets A, B, and D). Furthermore, in order to choose between sets, each percept has to be memorized at time 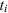 and retained while scrutinizing the other sets, posing a short-term memory demand.

Surface color is an informative cue to many object properties, such as the edibility of food. Figure 1 illustrates the use of color in the scenario of locating optimally ripe tomatoes in a market. Although this task is phenomenologically easy, it places considerable demands on visual information processing. First, notice how each group of tomatoes falls under a different illuminant: tomatoes in panels A and C are directly illuminated, while tomatoes in panels B and D are in shadow. To judge ripeness, the surface color of the tomatoes needs to be estimated, but this is a non-trivial task because the light reflected off the tomatoes confounds surface reflectance (which is the physical property of interest) and illumination. The insets in Figure 1 illustrate this confound: the differences in reflected light between sets of tomatoes may be caused by illumination or reflectance differences, or both. Once surface color and related object properties have been estimated for one set, these estimates have to be retained in memory while evaluating another set. This introduces a concomitant visual short-term memory demand. Although such joint employment of perceptual estimation and memory is a common feature of real-world color tasks, the two processes are rarely studied in the same paradigm. If perceptual and memory processes are independent, it is useful and convenient to study them separately. The fact that the independence assumption remains unverified, however, may lead to results overly specific to the study conditions; for instance, to a characterization of color memory that does not generalize to different color contexts.

**Figure 2:**
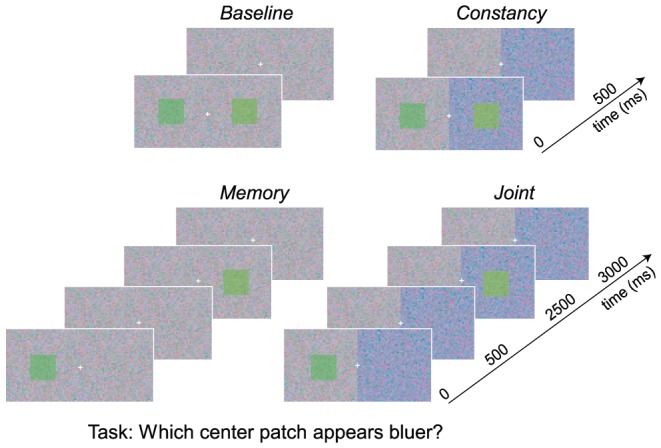
Four conditions were employed to test the independence of perception and memory. In the *baseline* condition, observers made hue judgments on a background whose average color was either gray (shown) or bluish. In the *constancy* condition, observers made hue judgments across an equiluminant color change in the background. In the *memory* condition, observers made hue judgments across a delay of 2 seconds. The background color was the same for both test and reference (either gray (shown) or bluish). In the *joint* condition, observers made hue judgments across both a change in background color and a delay. The observers’ task was always to select the stimulus that appeared bluer. The memory and joint conditions were also run with distractor stimuli displayed during the memory delay (see Figure S1). Note that the colors here were selected for illustration purposes, and are only approximate due to differences in display media.

The question of independence between surface color estimation and color memory is related to recent studies in several domains that demonstrate effects of short-term memory on perceptual estimates (e.g. perception of spatial frequency [1], bistable figures [2], motion direction [3], and structure-from-motion [4]). Our approach differs from the aforementioned work in that we are interested in how perception is affected by two concomitant demands — short-term memory and context change — that may or may not be independent.

The perceptual aspect of object identification by color has been extensively investigated under the rubric of color constancy [5]–[8]. This work has elucidated the task, display, and measurement characteristics that govern human color constancy in laboratory tasks. To briefly summarize, color constancy usually improves when cues to scene structure and illumination are added (e.g. [9]–[12]), and when tasks prompt observers to evaluate surface properties rather than proximal sensations (e.g. [13]–[16]). Even though studies in color constancy increasingly rely on realistic scenes and tasks, the effect of memory has received only scant attention; even in studies employing temporal color matching paradigms, pure short-term memory for color is usually not characterized.

Similarly, color memory for a single color context has been extensively characterized in a separate research program (e.g. [17]–[23]). Based on this work, there is broad consensus that color memory is more variable than perception, but whether this increased variability causes biases in color appearance is more controversial (see [24] for discussion). Because illumination or context color is not a variable of interest in this literature, it is agnostic about the relationship between constancy and memory processes.

A small number of studies have addressed some aspect of the relationship between color constancy and color memory [24]–[27]. Jin and Shevell (1996) showed that scene complexity aids color constancy only when there is a substantial memory delay between the training and match stimuli [25]. de Fez et al. (2001) found that a delay introduced into a simultaneous color constancy display increased the error in color matches [27], consistent with color memory studies for a single context [20]–[22]. Finally, Ling and Hurlbert (2008) compared successive color constancy to pure color memory and found that when pure memory errors were taken into account, successive color constancy was nearly perfect ([24]; also see [26]). There are two main differences between the previous work and our approach. First, we are focusing on short-term memory in the order of a few seconds, which is relevant for many natural color selection tasks, whereas previous studies used longer delays (often in the order of minutes). Second, the main aim of the present work is to test the independence of contextual and memory processing of color information. This requires a full-factorial design with memory and context manipulations, which has not been employed previously.

More generally, the relationship between appearance and the variability of both the sensory and memory representations that underlie appearance is emerging as a focus of interest in areas as diverse as color, temporal interval, line length, and speed estimation, as well as medical imaging [28]–[32]. Here, we test the independence of color constancy and short-term color memory by measuring both appearance and precision of color in conditions which place demands solely on perceptual constancy, solely on color short-term memory, or jointly on both constancy and memory. In addition to an independence test, this paradigm allows us to link our results to the broader scientific question of the relationship between appearance and precision.

## Results

The independence of color memory and color constancy was tested by measuring appearance and precision for hue in a 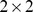 factorial design with constancy and memory manipulations. Figure 2 illustrates each main condition. In the *baseline* condition (top left), the reference and test stimuli were displayed simultaneously on identical (symmetric) backgrounds; in the *constancy* condition (top right), both stimuli were displayed simultaneously on different (asymmetric) backgrounds; in the *memory* condition, the stimuli were displayed with a delay of 2 s on symmetric backgrounds (bottom left); and in the *joint* condition, the stimuli were displayed with a delay of 2 s on asymmetric backgrounds (bottom right). The conditions were blocked with a counterbalanced order across observers. On each trial, the observer was asked to indicate which of two center patches appeared bluer. One patch was a reference which remained fixed across trials. The other patch was a test which was adjusted on each trial according to a staircase or method-of-constant-stimuli procedure (see Methods). Trials for three different reference stimuli, varying from yellowish-green to bluish-green, were interleaved in a block. The reference could appear either on the left or right on any given trial. These two cases were analyzed separately because the background colors in the constancy and joint conditions were fixed to gray on the left and blue on the right. From the left/right responses for each reference/background pairing, the probability of selecting each test hue as bluer than its reference was calculated. Psychometric functions (PMF) were estimated by fitting cumulative normals to the proportion-bluer data. The color appearance of a given reference was defined as the 50th percentile of the PMF, while the discrimination threshold was defined as the difference between the 75th and 50th percentile of the PFM. Precision was defined as the reciprocal of discrimination threshold. (See Methods for details.)

To accommodate both constancy and memory literatures, we adopt a slightly different terminology from what is common in color constancy studies. In general, color constancy refers to the degree of compensation for a (real or simulated) illumination change when estimating the surface color of a stimulus. We characterize this compensation by measuring the shift in color appearance caused by a difference in background color between the reference and test stimulus. We use the term *constancy bias* to denote this appearance shift, where more bias indicates better constancy (theoretically, there might be too much bias, i.e. overcompensation for the background difference, but this was not observed for any data set). We use the term *memory bias* to denote the appearance shift caused by short-term memory retention; and *joint bias* to denote the appearance shift caused by a combined background change and memory retention. Finally, we use color words such as *blue*, *gray*, *green*, etc. as convenient shorthands for stimulus hues.

### Hue appearance

**Figure 3:**
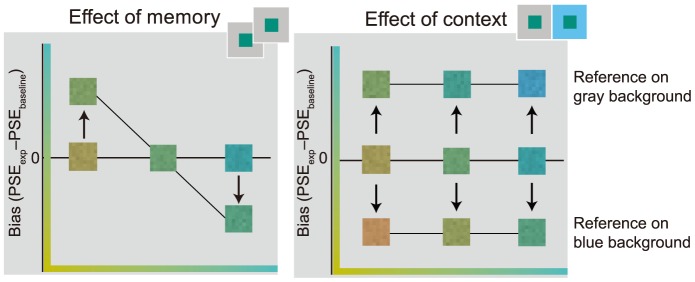
Expected memory and constancy biases. Illustration of predicted effects in the memory condition (left) and constancy condition (right). In both panels, the three reference hues are depicted on the x-axis; hue appearance bias is on the y-axis. Horizontal line indicates zero hue appearance bias. The left panel illustrates a potential central tendency bias for hue in the memory condition. If there were such a bias, the appearance of the extreme references should shift toward the middle stimulus value. This is indicated by the upward and downward arrows and by the colors of the insets. The right panel shows the color constancy prediction based on known color contrast effects. Consider the case when the reference is on the gray background (top half of panel): the test on the blue background should appear yellower than the reference, and thus it should be rendered physically “bluer” to match the reference in appearance. This is shown as an upward shift. In the case when the reference is on the blue background, the test on the gray background should appear relatively more bluish, and should be rendered physically “yellower” to match the reference. This is shown as a downward shift.

To give a sense of the expected effects of memory and context on hue appearance, and to familiarize the reader with our plotting conventions, predictions for the memory and constancy conditions are shown in Figure 3. The three reference hues are on the x-axis, and hue bias is on the y-axis. Zero bias, marked with a horizontal line, indicates no effect of a manipulation on hue appearance. The left panel shows a potential effect of memory on hue appearance; we anticipate a central tendency bias, whereby the appearance of the extreme references should be drawn toward the middle reference [29], [30], [33]. Specifically, the appearance of the most bluish and the most yellowish references should shift toward middle green, shown as a downward shift or an upward shift for the two references, respectively. The inset colors illustrate the perceptual effect of this shift. Similarly, the right panel shows a likely outcome for the constancy condition. Here background is used as a proxy for a simulated illuminant shift. Thus, having two different backgrounds can be expected to change the relative appearances of the reference and test [13], [14], [34]. Consider the case when the reference is on the gray background. A physically identical test on the blue background will be likely to appear yellower than the reference because of spatial color contrast. Thus, observers will be likely to require a bluer test to match the reference. A bluer test would result in an upward shift for all the reference hues (top of right panel). When the reference is on the blue background we expect an equal but opposite appearance shift (to yellower, bottom of right panel). The distance of the data from the horizontal zero-bias line indicates the magnitude of this compensation.

**Figure 4:**
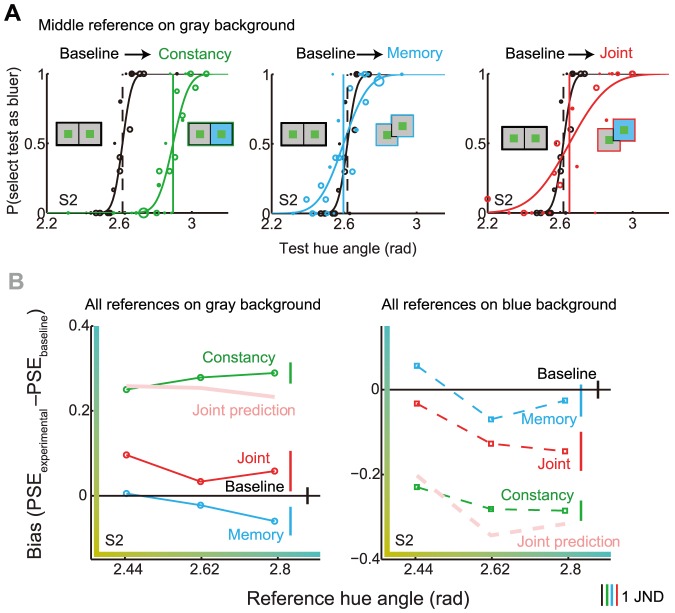
Estimating appearance and precision from psychometric functions. A) Example psychometric functions are shown for a representative observer for the middle green reference. Within each panel, the cumulative normals show the probability of selecting the test stimulus as bluer as a function of test hue (larger hue angles correspond to bluer hue appearance). The black curves in all panels show the same baseline data. The colored curves show the data from the labeled condition. The vertical dashed lines show the reference hue, that is, the veridical match, whereas the solid vertical lines show the actual matches (PSEs). Bias is defined as the difference between the dashed and solid lines. The slopes of the psychometric functions indicate precision. B) The appearance shifts (bias) are shown for each of the three reference stimuli in each condition (labeled). The right-hand panel shows data for those trials where the reference stimulus was on the gray background and the test on the gray (baseline, memory), or on the blue background (constancy, joint). The left-hand panel shows data from those trials where the reference was on the blue background and the test on the blue (baseline, memory), or on the gray background (constancy, joint). As the bias in each condition was calculated relative to the PSE in the baseline condition, the baseline bias (black horizontal line) is always zero. The vertical line segments on the right indicate threshold hue angles (JND’s) in each condition averaged over reference stimulus. Longer lines indicate decreased precision. Figure S2 shows data for all observers.

Figure 4 demonstrates for one representative observer how appearance and discriminability were derived from the raw data. Example psychometric functions are shown in Figure 4A for the constancy (left), memory (middle), and joint conditions (right). The data for these functions were derived from trials where the reference was middle green and was displayed on the gray background; the test was displayed on the gray background (memory condition) or on the blue background (constancy and joint conditions). Some of the more robust effects, which we analyze more closely below, are readily seen here. For instance, making hue matches across a change in background in the constancy condition had a large effect on color appearance but little effect on precision: the location of the psychometric function in the constancy condition was shifted compared to the baseline condition, but the slope remained the same. Memory, on the other hand, seemed to decrease precision relative to baseline, shown by the shallower slope of the blue curve, but did not seem to have a large effect on color appearance, shown by the overlap of the curves. In the joint condition, there were often concomitant changes in appearance and precision.

From all measured psychometric functions, we extracted the shifts in hue appearance for the three reference stimuli in all four conditions, and plot these for observer S2 in Figure 4B. The left-hand panel shows the data from the trials where the reference was displayed on the gray background, and the test was displayed either on the gray background (when the backgrounds were symmetric) or on the blue background (when the backgrounds were asymmetric). The right-hand panel shows the data from the trials where the reference was displayed on the blue background, and the test was displayed either on the blue (symmetric) or on the gray (asymmetric) background. Positive biases indicate an overall shift toward bluer hues, and negative bias indicates an overall shift toward yellower hues.

First, consider the constancy condition (green lines). As shown in Figure 3, a color constant observer would show an overall shift upward or downward to compensate for the simulated illumination change. When the reference was on the gray background, the shift was in the expected direction: the hue angle of the test on the blue background was adjusted upward to match the reference. When the reference was on the blue background, the shift was in the opposite direction and had roughly the same magnitude as the bias in the left-hand panel.

Next, we turn to the memory condition (blue lines). For this observer, there was a small overall shift toward yellower hues in memory, but the shift was not the same for all references; the bias across reference hue had a negative slope, indicative of a central tendency bias toward the middle reference. That is, the most yellow reference (leftmost on x-axis) was remembered as less yellow (upward shift relative to middle reference) and the least yellow reference (rightmost on x-axis) was remembered as more yellow (downward shift relative to middle reference). This tendency was clear for the gray reference background (left panel) and somewhat present for the blue background (right panel).

To evaluate the joint effect of constancy and memory on appearance, we may compare the joint data to an independence prediction that is derived from pure memory and constancy biases (see Methods). A significantly smaller or larger joint bias than the prediction would suggest an interaction between constancy and memory. For this observer, comparison of the joint bias to the independence prediction (compare thin red to thick pink lines) reveals that the joint bias was much smaller than predicted; indeed, it was on average only 30% of the predicted bias for both reference backgrounds. The other observers replicated the main features of the data (see Figure S2).

The vertical line segments next to each data set in Figure 4B indicate one threshold unit (JND) for a given condition, averaged over reference. Comparing the length of the line segments to bias magnitude serves to give an idea of the perceptual salience of the biases in each condition. The memory bias was not perceptually large, although for some references it was clearly discriminable. On the other hand, the constancy bias was highly noticeable, as was the difference between the constancy and joint biases.

**Figure 5:**
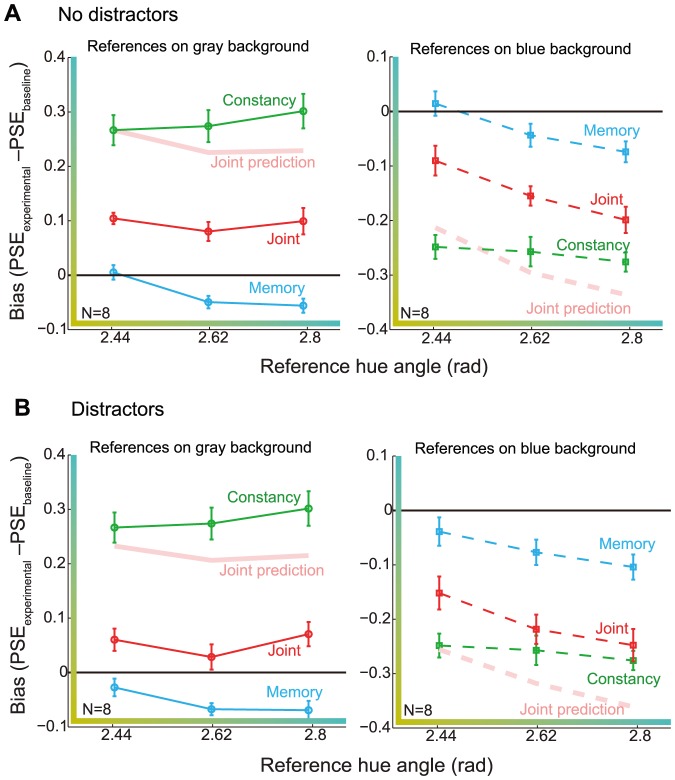
Independent color and memory biases do not predict joint bias. A) Bias averaged over eight observers is shown for each experimental condition and reference hue angle. The memory and joint conditions had a blank delay interval. The left-hand panel shows data from the trials when the reference was on the gray background. The right-hand panel shows data for the trials when the reference was on the blue background. Error bars here and in following figures indicate 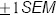. B) Average bias is shown for the memory and joint conditions where distractors were displayed for 500 ms in the middle of the 2 s delay. Distractor hues were selected from a normal distribution centered on smaller (yellower-appearing) hue angles relative to reference hue. Constancy data are replotted from A). Joint prediction was calculated based on the constancy data from A) and memory data with distractors.

Figure 5A shows appearance data averaged over all observers. The left and right-hand panels show data for the gray and blue reference backgrounds, respectively. As expected, hue matches in the constancy condition were shifted in the direction of color constancy. The memory bias was also evident in the mean data, and exhibited a negative slope across reference stimuli in addition to an overall downward shift. The central tendency bias, as defined by the negative slope of the regression line fitted to the memory data across references, was present in all but one individual data set (slope mean 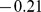, range 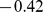 – 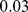; one-tailed sign test 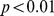). The overall downward shift in the memory bias may be a response bias due to task wording.

**Figure 6:**
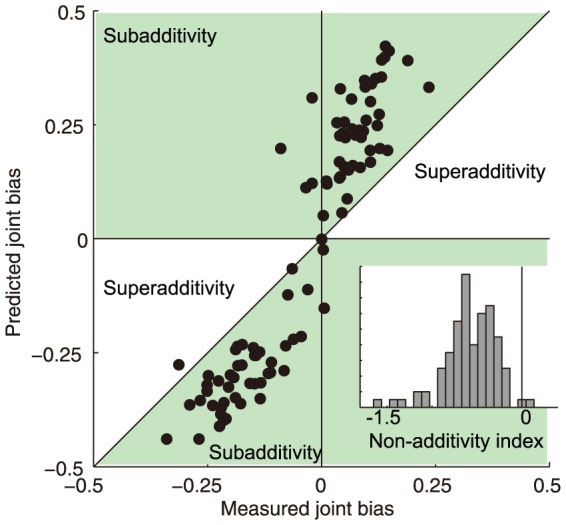
Constancy and memory biases are subadditive. Predicted joint bias is plotted against measured joint bias for all observers and reference/background pairs. Positive diagonal indicates full additivity, or independence, between constancy and memory. Green shaded areas indicate subadditivity. The inset shows a histogram of non-additivity indices. In the inset, vertical line indicates full additivity; negative values indicate subadditivity.

Most importantly, the joint bias was on average much smaller than the prediction derived from independent constancy and memory biases. This subadditivity was a pervasive feature of the entire data set. Figure 6 plots predicted against measured joint bias for each observer and reference stimulus. If there was full additivity, the data points would fall close to the diagonal. This is clearly not the case, as almost all data points fall in the subadditivity region indicated with green shading; on average, measured joint bias was 42% of predicted joint bias. We quantified subadditivity with a non-additivity index, where zero indicates full additivity, negative values subadditivity and positive values superadditivity. The inset in Figure 6 shows a histogram of non-additivity indices calculated for each observer, reference stimulus, and reference background. The non-additivity indices were significantly subadditive (mean NI: 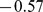; one-tailed 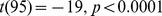).

The memory and joint data shown in Figures 4B and 5A were collected with a blank screen between the reference and test displays. To test whether increased memory load would affect the pattern of biases, we ran additional memory and joint conditions with distractors. These conditions were otherwise identical to the delay conditions depicted in Figure 2, but instead of a blank screen, distractor stimuli were displayed for 500 ms in the middle of the 2 s delay (see Methods and Figure S1). These data are shown in Figure 5B; the constancy data are replotted from Figure 5A. Adding distractors mainly caused the memory and joint biases to shift downward (toward yellow), but the pattern of biases across reference stimuli was similar. In a mixed-effects 3-way ANOVA with distractors (present/absent) and reference hue as fixed effects and subject as random effect, there was a main effect of distractors (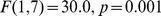) and reference (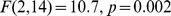) but no interaction between distractors and reference (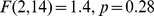). The shift toward smaller hue angles probably reflects the fact that the distractors were sampled from a normal distribution approximately 1.5 JND’s toward smaller (“yellower”) hue angles from each reference, and thus drew the mean hue of all displayed stimuli toward yellower-appearing hues.

### Hue precision and its relationship to appearance

In addition to affecting color appearance, experimental condition could also affect sensitivity to color differences. Indeed, the psychometric functions for observer S2 in Figure 4A were steeper in the baseline and constancy conditions than in the memory and joint conditions, indicating a difference in precision. To quantify this effect, we examined the thresholds derived from psychometric functions, where larger thresholds indicate decreased precision.

**Figure 7:**
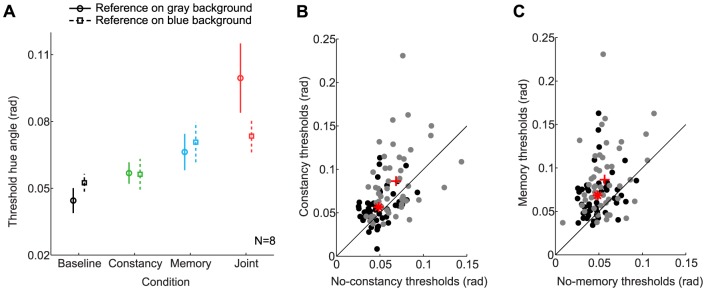
Constancy and memory demands affect precision, quantified by discrimination thresholds. A) Average threshold hue angles are shown for each condition, averaged over reference hue for the gray reference background (circles and solid lines) and blue reference background (squares and dashed lines). B) The main effect of context on thresholds is shown by plotting thresholds from the asymmetric background conditions (constancy and joint) against symmetric background conditions (baseline and memory). Each data point is for one observer, reference, and reference background. No-memory conditions are indicated in black (baseline vs. constancy, average  =  red asterisk) and memory conditions are indicated in gray (memory vs. joint, average  =  red plus). C) The main effect of memory on thresholds is shown by plotting thresholds from the memory conditions (memory and joint) against no-memory conditions (baseline and constancy). Symmetric background conditions are indicated in black (baseline vs. memory, average  =  red asterisk) and asymmetric background conditions are indicated in gray (constancy vs joint, average  =  red plus).

Thresholds for each condition, averaged over references and observers, are shown in Figure 7A. Thresholds in the baseline condition were on average lowest, and were progressively higher in the constancy, memory, and joint conditions. The separate effects of constancy and memory on thresholds can be gleaned from Figures 7B and C, respectively. In both panels, each data point is for one observer and one reference/background pair. Figure 7B shows that constancy had a small effect on precision: thresholds were on average 1.3 times higher in the constancy conditions than in the no-constancy conditions. Figure 7C illustrates the effect of memory: thresholds were on average 1.6 times higher in the memory conditions compared to the no-memory conditions. However, there was substantial interindividual variability in thresholds, especially in the joint condition (see e.g. red symbols in 7A). We conducted a 4-way mixed-effects ANOVA to test the effects of constancy and memory on thresholds, where constancy, memory, and reference background were entered as fixed effects, and subject as a random effect. The main effect of memory was significant (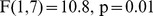), as was the main effect of constancy (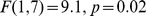). There was no significant main effect of reference background (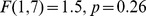), nor significant interaction between memory and reference background (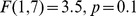). The interaction between constancy and reference background was significant, however, indicating that constancy affected thresholds more when the reference was on the gray background (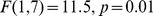). Finally, as indicated by the mean data in Figure 7B and C — all red symbols are above the unity line — there was no interaction between constancy and memory manipulations on thresholds (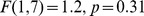).

**Figure 8:**
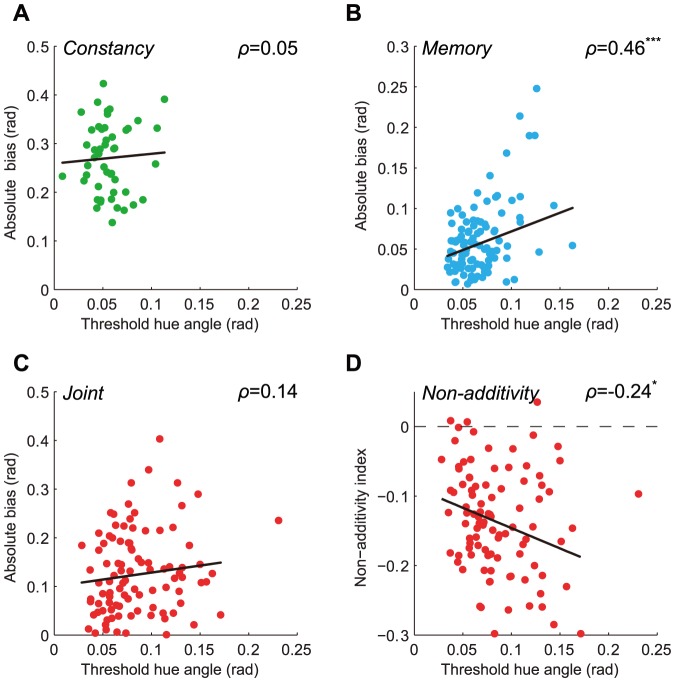
Decreased precision is related to memory bias and subadditivity. A-C) The absolute values of constancy (A), memory (B), and joint (C) bias are plotted against thresholds in the same conditions for all observers and reference/background pairs. D) Non-additivity index is plotted against joint thresholds for each observer and reference/background pair. Negative index values indicate subadditivity. In all panels, lines show linear regression fits with slope and intercept. Correlation coefficients (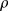) are given in each panel; * indicates significance at the 0.05 level, and *** at the 0.001 level. In C) and D), one threshold was excluded from analysis as an outlier (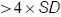; data point visible at the edge of both plots).

Next we investigated the relationship between appearance and precision. The underlying assumptions about the mechanisms responsible for thresholds influence alternative predictions. For example, if increased thresholds reflect the conjunction of two independent sources of variable sensory information, as is possible with the two independent backgrounds in the constancy condition, we might not expect any relationship between thresholds and the magnitude of appearance bias. If, on the other hand, higher thresholds indicate decreased reliability of sensory information, as they might in the case of memory, and if that decreased reliability affects sensory decisions, then we might expect to find a systematic relationship between thresholds and bias size. Figure 8 shows the relationship between bias and thresholds for the constancy (A), memory (B), and joint (C) conditions. Consistent with the prediction for two independent sources of variability, there was no correlation between thresholds and bias in the constancy condition (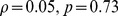). In the memory condition, however, there was a moderate correlation between thresholds and bias, consistent with the hypothesized relationship between decreased reliability of sensory information and perceptual bias (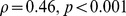). Finally, there was no correlation between thresholds and bias in the joint condition (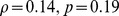). This was presumably due to the joint bias being a combination of constancy and memory biases, where only the memory bias was significantly related to thresholds. There was a significant relationship between subadditivity of the joint matches and joint thresholds, however: less precise matches were more subadditive (Figure 8D, 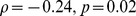).

## Discussion

The present study shows that short-term color memory modulates the effect of context on color appearance. This result is incongruent with the implicit assumption in color perception and color memory research that constancy and memory processes are independent, and thus has implications on the generalizability of traditional color constancy and color memory studies. Existing color appearance models, such as ones relying on contrast coding between a target stimulus and its context (e.g. [35]) can conceivably account for our results in the simultaneous conditions, but it is not clear how these models would incorporate memory effects. However, the varying relationship between precision and appearance across conditions suggests interpreting the results in a Bayesian framework. The Bayesian approach allows memory to influence perceptual judgments by altering the reliability of the sensory signal. Interpreting the joint effects of color context and memory in this framework seems a natural step, since Bayesian models can account successfully for color constancy phenomena without memory [28], [36], [37], and for memory effects without constancy [29], [30].

In Bayesian models of perception, an estimate of an object property is formed by combining a noisy sensory measurement with prior information on the probability of different property values [38]. In the case of surface color perception, the goal is to estimate surface reflectance from a noisy measurement of the incoming light signal. In this context, the *likelihood function* describes the probability of a particular sensory measurement given reflectances and illuminants in the world. Following the Bayes’ rule, the *posterior probability* of a particular reflectance is given by combining its likelihood (given the measurement) with the prior probability for that reflectance. The width of the likelihood function reflects the noise in the measurement; thus, the noisier the signal (and thus the broader the likelihood), the more effect the prior will have on the perceptual estimate.

Our conceptual framework makes the following assumptions based on previous literature: 1) observers are estimating (explicitly or implicitly) both surface reflectance and illumination [28], [36], [37]; on each trial, they pick the stimulus whose inferred reflectance is relatively more “bluish” 2) the background color dominates the observers’ illumination estimate based on its spatial extent [28]; 3) observers have available priors for reflectance and illumination [28], [36]; 4) both priors can be updated based on reflectance and illumination estimates in a block of trials [29], [30], [39]–[41]. In the following, we interpret our results in this framework and show that the framework qualitatively accounts for the patterns of biases across experimental conditions.

In the baseline condition, the hue estimates were unbiased. This is an expected result if we assume that the measurements for the simultaneously presented reference and test were equally noisy. Thus, the reference and test likelihoods had similar widths, which caused the prior to have the same effect on both estimates. Furthermore, the inferred illumination for both reference and test was the same, thus causing no context bias.

In the memory condition, there was a small but significant hue bias toward the middle reference hue. There was also an increase in thresholds relative to baseline. The correlation between thresholds and bias indicates that noisier memory representations were more biased. This would be expected if the likelihood for the memorized reference were broader than for the test, and if the reflectance prior were non-uniform. Indeed, we suggest that the reflectance prior was centered on the mean estimated reflectance in a block of trials. Combining a non-uniform prior with the broader reference likelihood would cause the reference estimate to be drawn toward the prior, inducing a central tendency bias [29], [30]. This conjecture is further supported by the effect of distractors on reflectance estimates: in the delay blocks where yellow-biased distractors were displayed during the retention interval, estimates were drawn toward yellower-appearing hues. This was presumably due to the reflectance prior being affected by the distractors.

In the constancy condition, there was a uniform hue bias for all references. In other words, the asymmetric background caused a hue appearance difference between reference and test. This is consistent with different illuminant estimates for the reference and test, as follows. Suppose that the stimulus on the gray background was perceived to be under a relatively neutral illumination, while the stimulus on the blue background was perceived to be under a relatively bluish illumination. In this case, two physically identical stimuli would be inferred to have different reflectances: the stimulus under the bluish illumination would be inferred to have a relatively “yellower” reflectance than the stimulus under the neutral illumination (see [42] for an analogous explanation of simultaneous color contrast). This would cause a bias in the estimates whose sign depended on whether the reference was on the gray or on the blue background. There should, however, be no bias toward the surface prior because the likelihoods for the simultaneously presented reference and test would be equally broad. Indeed, the bias was uniform across references. Thresholds did increase from the baseline to the constancy condition, but contrary to the memory condition, threshold size was not related to the magnitude of constancy bias. This is consistent with observers making two independent illuminant estimates instead of one, which may have introduced more noise in the posterior estimate.

How can this framework account for the interaction between constancy and memory in the joint condition? Recall that context had less effect on color appearance in the joint condition compared to the simultaneous constancy condition. In other words, the delay between reference and test interval caused the two backgrounds to become functionally more similar. This is consistent with the observer estimating *both* the reflectance of the center patch *and* the color of the illumination, as follows. Let us assume that the mean of the illuminant prior was at the average estimated illumination from the gray and blue backgrounds. Similarly to the pure memory condition, the likelihoods for the center patch and background in the reference interval would become broader due to memory retention. This would cause a shift in both estimates toward their respective priors. Crucially, the shift of the reference illuminant estimate toward its prior would cause the reference illuminant to be more perceptually similar to the test illuminant than in the simultaneous constancy condition. A smaller perceptual difference should lead to less compensation than predicted from the veridical difference. This would manifest as less color constant matches in the joint condition, and thus subadditivity between constancy and memory. The positive correlation between joint thresholds and subadditivity supports this interpretation: those observers who had more variability in their joint matches presumably relied more on their priors, leading to more subadditive joint matches.

Our conceptual framework is agnostic about the neural mechanisms underlying the interaction between contextual processing and short-term memory, but recent work in other stimulus domains suggests that such interactions might have an early cortical locus. Several physiological and fMRI studies have shown that early sensory networks play an important role in the retention of feature-specific short-term memories [43]–[47]. In line with this, recent behavioral studies have demonstrated effects of short-term memory on perceptual estimates for spatial frequency, structure-from-motion, and motion direction [1]–[4]. Furthermore, BOLD activation in early sensory networks has been found to be modulated by the use of prior information in perceptual decisions [48]–[50]. Neural or BOLD measurements for the interaction between color perception and memory are not available, but early cortical networks for color perception are relatively well-characterized and thought to overlap with networks for other low-level stimulus features [51]. Thus, it is conceivable that the interaction between color memory and contextual color processing (via learned expectations about hue) might take place in these early cortical color networks.

In conclusion, short-term memory influences contextual color processing in a way that is consistent with the observer taking prior information into account in varying degrees, depending on the variability in the sensory signal. This interaction suggests a close connection between perceptual and memory processing of color, and demonstrates the importance of jointly studying perceptual and memory processes when the goal is to understand real-world tasks.

## Methods

### Ethics statement

Written informed consent was obtained from all study participants prior to their enrollment in the study. The participants were compensated at $10/hour. The experimental protocol adhered to the Declaration of Helsinki, and was approved by the Rutgers University Institutional Review Board.

### Participants

Eight naive participants (2 males; mean age 26, SD 9.5) observed in the study. Six participants repeated the conditions three times for 10 hours of observation; two participants repeated the conditions twice for seven hours of observation. All participants had normal or corrected to normal visual acuity and normal color vision as assessed by the Ishihara color plates.

### Apparatus

Stimuli were displayed on a calibrated CRT monitor (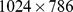 pixels/24
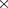
18 deg; 85 Hz) with a 10-bit intensity resolution per color channel via the Datapixx box (VPixx Technologies, Inc.). The monitor was calibrated once a month with standard methods [52].

MGL functions (URL: http://gru.brain.riken.jp/doku.php/mgl/) were used for stimulus display and data collection in Matlab (Mathworks, Inc.).

### Stimuli and procedure

Observers viewed the display from a 94 cm distance, controlled with a chin rest. On each trial, the observer saw two stimuli, one reference and one test. The stimuli subtended 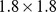 degrees of visual angle and were displayed 3 degrees on the left or the right of a central fixation cross for 500 ms. The two stimuli were displayed either simultaneously or with a 2 s delay, depending on condition (see below). The observer’s task was always to select the stimulus that appeared bluer by pressing the corresponding button (left/right). The left/right locations of the reference and test were randomized on each trial. In delay conditions, the reference was always displayed in the first interval.

Three reference hues chosen from the equiluminant hue circle in CIELAB space were employed: yellowish-green, green, and bluish-green (see Table S1 for CIE chromaticities). Relatively similar reference hues were selected to avoid verbal labeling strategies [53]. Test hue was controlled with a staircase or a method-of-constant-stimuli (MOCS) procedure (see below). The saturation and luminance (15 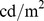) of all displayed stimuli were kept constant throughout the experiment.

Constancy and memory demands were manipulated in a 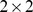 factorial design (see Figure 2). The constancy manipulation consisted of presenting stimuli on either symmetric (no-constancy) or asymmetric (constancy) backgrounds. The symmetric backgrounds were either gray or blue in color appearance (see Table S2 for background chromaticities). The asymmetric backgrounds were split vertically in the middle of the display into gray and blue fields. The gray field was always on the left, and the blue field was always on the right. Changing background as a proxy for a changing illuminant is a standard method for measuring constancy in CRT displays [12], [13], [54], [55]. Memory was manipulated by presenting stimuli simultaneously (no memory) or with a 2 s delay (memory). The four resulting conditions are illustrated in Figure 2: baseline (symmetric backgrounds, no delay), constancy (asymmetric backgrounds, no delay), memory (symmetric backgrounds, 2 s delay), and joint (asymmetric backgrounds, 2 s delay).

The memory and joint condition had two variants: without and with distractors. In the conditions without distractors, shown in Figure 2, the 2 s interval between the reference and test was blank (backgrounds and fixation cross were always present). In the conditions with distractors (see Figure S1), two stimuli with the same dimensions and locations as the reference and test were displayed in the middle of the delay interval for 500 ms. The hues of the distractors were sampled from a normal distribution whose mean was 1.5 average just-noticeable-differences (0.2 radians) away from the reference hue toward smaller hue angles.

Both the stimuli and backgrounds consisted of a checkerboard texture (check size 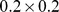 deg). The luminance of the stimulus checks was perturbed around the mean display value of 15 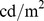. The checks in the background were perturbed in both luminance and chromaticity around the mean xyY values given in Table S2. The mean luminance and check size of the stimulus and background textures were identical to maximize color induction from the background [56].

A two-stage procedure was employed to obtain reliable threshold estimates. In the first part of the experiment, psychometric functions in each condition were measured with a staircase procedure. In other words, the test hue on each trial was determined by the response on the previous trial according to a given decision rule. Four interleaved staircases tracked roughly the 20th and 80th percentiles of each psychometric function, with starting points both above and below the reference hue. Each staircase had 20 trials. Staircases for the three different reference stimuli and for the two reference backgrounds were interleaved, resulting in 240 trials per block when the background was uniform (baseline, memory), and 480 trials when the background was asymmetric (constancy, joint). Cumulative normals were fitted to the proportion-test-selected-as-bluer data in each condition. Based on these fits, the test hues that corresponded roughly to 0 and 100% selection probability were chosen as endpoints for the next phase of the experiment. In this phase, data were collected for five test levels evenly distributed between the endpoints with the method of constant stimuli (MOCS). Specifically, on each trial the test hue paired with a given reference was randomly selected from the five predetermined values. Each test level was repeated 10 times during one block, with the three references and two backgrounds interleaved. After finishing one complete MOCS run, the range was adjusted when necessary, after which 10 more repetitions for each level were collected.

The six conditions (baseline, constancy, memory blank, memory with distractors, joint blank, joint with distractors) were blocked, and their order in each phase of the experiment was counterbalanced across observers. Each experimental session contained one or more blocks and lasted between 45–60 minutes.

### Data analysis

#### Psychometric function fitting.

The data from the staircase and MOCS run were pooled for analysis. From the pooled data, we calculated the probability at each test hue of selecting the test stimulus as bluer than the reference. Psychometric functions (PMFs) were estimated by fitting cumulative normals to the proportion-test-bluer data with the Psignifit package [57], [58]. Color appearance of a reference was defined as the 50th percentile of the PMF. This point denotes the test hue which is perceptually indistinguishable from the reference hue (point of subjective equality, PSE). Bias in each condition was defined as 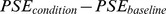. To quantify precision, we defined hue discrimination thresholds as the hue angle spanned by the difference between the 75th and 50th percentile of the PMFs.

As noted, on each trial observers selected the stimulus that appeared bluer. It has been demonstrated that this kind of binary task, where the two choices lie along a one-dimensional continuum, may induce response biases in one or the other direction that may be distinct from perceptual biases (see [59] for discussion). For example, even though the observer might perceive stimuli as identical in appearance, the wording of the task ( “which one is bluer” vs. “which one is yellower”) might drive the responses toward one end of the continuum. Although we cannot be certain that observers did not display response biases of this sort, we note that such response biases would presumably be present in all conditions since the wording of the task remains the same. We operationally define the *perceptual* bias as the difference between the PSE in an experimental condition and the baseline condition; thus, any response bias should be subtracted out.

#### Independence analysis.

The pure memory effect is characterized by the shift in appearance due to memory. The pure constancy effect is characterized by the shift in appearance due to constancy. If constancy and memory act independently, the bias in the joint constancy-and-memory condition should be a fully additive combination of the separate effects of memory and constancy. This consecutive, independent effect of memory and constancy on color appearance can be derived by first applying the memory effect on the three references, and consequently applying the constancy effect to the memory-biased reference hues. This is equivalent to taking the memory-biased hue matches as new reference stimuli and deriving constancy matches to those. As we did not measure constancy matches to reference stimuli defined by the memory-biased hues, we interpolated from the existing constancy data, assuming constancy to be a homogeneous function of reference hue in this range. Formally, denote the reference hue by 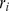, the memory match to each 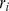 by 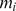, and the constancy match to each 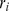 by 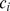, where 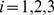 for the three reference hues. If perception and memory are independent, the joint match, 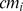 for a given 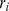 should be the constancy match to 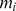 rather than to 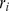. To obtain constancy matches to the reference hues defined by 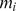, we interpolated from the measured 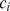. In the few cases where 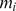 were outside the 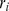 range, we extrapolated; the maximum departure from the range was 4%.

After deriving the predicted joint matches, independence in the measured joint matches was quantified with a non-additivity index 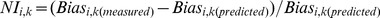, where 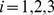 for reference stimulus and 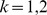 for the two reference backgrounds. A non-additivity index of zero indicates full additivity; negative values indicate subadditivity, and positive values superaddivity.

#### Statistical analyses.

Effects of the various experimental manipulations on hue appearance and precision were tested with mixed-model ANOVAs with the within-subjects manipulations as fixed effects and subject as a random effect. All interaction terms were entered into the model. In addition, t-tests and non-parametric sign tests were employed where appropriate. Details pertaining to each analysis are given where the results are reported.

## Supporting Information

Figure S1 **Delay conditions with distractors.** Memory (left) and joint (right) conditions were run without and with distractors in separate blocks. Here, the blocks with distractors are shown. Distractor hues for the reference and test locations were sampled from a normal distribution on each trial. The mean of the distribution was approximately 1.5 JND’s toward smaller (“yellower”) hue angles from the reference hue on a given trial. The distractor colors depicted here have been exaggerated for demonstration purposes. Distractors were shown for 500 ms in the middle of the delay period. The timing of the reference and test stimuli was identical to the conditions without distractors (see Figure 2 of the main text).
(EPS)

Figure S2 **Appearance shifts in each condition are plotted for individual observers in panels.** In each panel, bias hue angles in each condition are plotted for each of the three reference stimuli and for the two reference backgrounds. Conditions are indicated as follows: constancy (green), memory (blue), and joint constancy-and-memory (red). Independence predictions for the joint condition are shown with thick pink lines. Horizontal lines indicate zero bias relative to baseline. Data for the gray reference background are plotted in circles and solid lines; data for the blue reference background are plotted in squares and dashed lines. Threshold hue angles averaged over reference stimuli are indicated with line segments on the right of each panel. Longer lines indicate higher thresholds and decreased precision.
(EPS)

Table S1 Mean CIE 1931 xyY and CIE L*a*b* values of the three reference stimuli.
(PDF)

Table S2 **Mean CIE 1931 values and correlated color temperatures of the two backgrounds.**
(PDF)
