# Supplementary material for: Short-Term Memory Affects Color Perception in Context
Source: PLoS One. 2014 Jan 27;9(1):e86488. doi: 10.1371/journal.pone.0086488 (PMC3903542; doi:10.1371/journal.pone.0086488)
Supplement: Table S1 — Mean CIE 1931 xyY and CIE L*a*b* values of the three reference stimuli. (PDF) [file pone.0086488.s003.pdf]

Table S1: Mean CIE 1931 xyY and CIE L*a*b* values of the three reference stimuli. Y and L* values correspond to the mean of the normal distribution from which the luminances were sampled for each check in the stimulus. L*a*b* values have been calculated based on the neutral background (see Table S2).

Color appearance	hue angle (rad)	x	y	Y (cd/m^2)	a*	b*	L*
Yellowish-green	2.44	0.314	0.399	15	-30.6	25.7	100
Green	2.62	0.299	0.391	15	-34.6	20	100
Bluish-green	2.79	0.286	0.380	15	-37.6	13.7	100
