# Supplementary material for: Short-Term Memory Affects Color Perception in Context
Source: PLoS One. 2014 Jan 27;9(1):e86488. doi: 10.1371/journal.pone.0086488 (PMC3903542; doi:10.1371/journal.pone.0086488)
Supplement: Table S2 — Mean CIE 1931 values and correlated color temperatures of the two backgrounds. (PDF) [file pone.0086488.s004.pdf]

Table S2: Mean CIE 1931 values and correlated color temperatures of the two backgrounds.

Color appearance	x	y	Y	K
Neutral	0.313	0.329	15	6500
Bluish	0.279	0.391	15	10000
